# Delayed Response of Lake Area Change to Climate Change in Siling Co Lake, Tibetan Plateau, from 2003 to 2013

**DOI:** 10.3390/ijerph121113886

**Published:** 2015-10-30

**Authors:** Guihua Yi, Tingbin Zhang

**Affiliations:** 1 Institute of Mountain Hazards and Environment, Chinese Academy of Science, Chengdu 610041, China; E-Mail: yigh@cdut.edu.cn; 2 College of Administrative Science, Chengdu University of Technology, Chengdu 610059, China; 3 College of Earth Sciences, Chengdu University of Technology, Chengdu 610059, China; 4 Key Laboratory of Geoscience Spatial Information Technology, Ministry of Land and Resources of the P.R. China, Chengdu 610059, China

**Keywords:** lake, climate change, delayed response, MODIS, grey relational analysis, Siling Co, Tibetan Plateau

## Abstract

The Tibetan Plateau is a key area for research on global environmental changes. During the past 50 years, the climate in the Siling Co lake area has become continuously warmer and wetter, which may have further caused the increase in Siling Co lake area. Based on the Siling Co lake area (2003 to 2013) and climate data acquired from the Xainza and Baingoin meteorological stations (covering 1966 to 2013), we analyzed the delayed responses of lake area changes to climate changes through grey relational analysis. The following results were obtained: (1) The Siling Co lake area exhibited a rapid expansion trend from 2003 to 2013. The lake area increased to 2318 km^2^, with a growth ratio of 14.6% and an annual growth rate of 26.84 km^2^·year^−1^; (2) The rate of air temperature increase was different in the different seasons. The rate in the cold season was about 0.41 °C per ten years and 0.32 °C in hot season. Precipitation evidently increased, with a change rate of 17.70 mm per ten years in the hot season and a slight increase with a change rate of 2.36 mm per ten years in the cold season. Pan evaporation exhibited evidently decreasing trends in both the hot and cold seasons, with rates of −33.35 and −14.84 mm per ten years, respectively; (3) An evident delayed response of lake area change to climate change is observed, with a delay time of approximately one to two years.

## 1. Introduction

The Fifth Assessment Report of the Intergovernmental Panel on Climate Change (IPCC) shows that the average global surface temperature increased by 0.89 °C (0.69 °C to 1.08 °C) from 1901 to 2012 [1]. Represented by global warming, climate change has become an important environmental problem worldwide [2,3]. Along with the South and North Poles, the Tibetan Plateau has become a hotspot for research on global changes. Climate change in the Tibetan Plateau is not only an important part of global climate change, but it also acts as the “trigger” and “amplifier” of global climate fluctuations [4]. Climate change may cause water resource changes could lead to water resource redistribution in time and space and total water resource changes, which could significantly affect the natural ecosystem and the survival of human beings. As the largest and highest plateau lake region, the Tibetan Plateau has thousands of lakes of different sizes. Most of these lakes are still in their natural conditions and free from human activities, so they can truly reflect climate conditions [5]. With lakes acting as sentinels, integrators, and regulators of climate changes [6,7,8], many scholars have conducted research on the relationship between Tibetan Plateau lakes and climate changes. Recent research has shown that the lakes in the Nagqu and Kekexili Regions have generally expanded, whereas the lakes in the source region of the Yellow River in general exhibited evident shrinkage [9]. Among the lakes in the Tibetan Plateau, Siling Co is the largest. In recent years, the climate in Siling Co lake area has become warmer and wetter. The main reasons for the expansion of Siling Co lake area include increasing air temperature, surface temperature, and annual precipitation, decreasing evaporation, and melting glaciers and permafrost [10,11,12,13,14,15].

Previous analyses of the responses of lake area changes to climate changes generally analyzed qualitatively the influencing factors of climate changes [11,14,16,17,18] or linear fitting of climate data and lake area in the corresponding period [10,12,13,19]. Qualitative analysis can determine the deterministic response trend between lake area changes and climate changes, but the quantity of responses is unclear and indefinite. Previous linear fitting analyses were based on the synchronous responses of lake area changes to climate changes. In his research on the responses of glacier terminus changes to climate changes, Ding reported that the delayed response of the fluctuation of small glaciers (length ≤ 5 km) to climate changes is approximately two years [20]. There were also delayed responses of lake area changes to climate changes. However, only a few quantitative studies on the delayed response of lake area changes to climate changes have been conducted. In this study, we adopted grey relational analysis (GRA) [21] to determine the delayed response of the Siling Co lake area changes to climate changes and obtain the time scale influenced by air temperature, precipitation, and evaporation on similar lake areas.

## 2. Study Area

**Figure 1:**
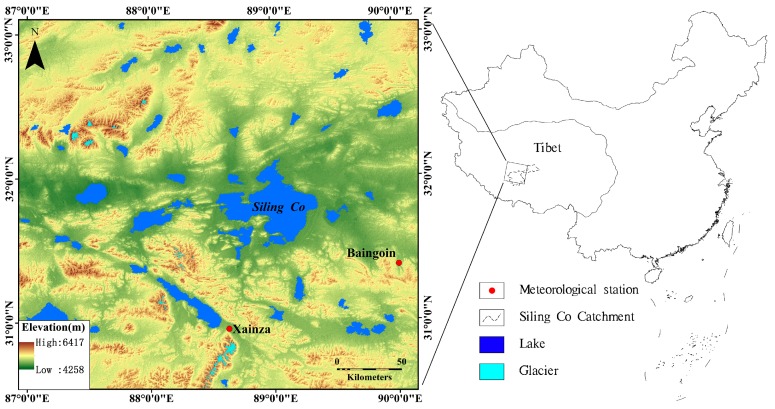
Location of the study area and meteorological stations in China.

Siling Co is located at the junction of three counties, namely, Xainza, Baingoin, and Nyima. In October 2010, the elevation of Siling Co was 4542.5 m and its area was approximately 2323.6 km^2^ [13]. It is the largest salt water lake in Tibet (Figure 1). The Siling Co Basin area is approximately 45,530 km^2^ and is the largest inland lake river system in Tibet. In the basin, numerous rivers and lakes are interconnected and form a closed inland lake group. In addition to Siling Co, 23 other small lakes, such as Geren Co, Wuru Co, and Co-e, are located in the area. The main rivers flowing into Siling Co include the Za’gya Zangbo, Zagen Zangbo, and Bogcang Zangbo. Za’gya Zangbo originates in the Tanggula Mountains and inflows from the north bank of Siling Co. It is the longest inward flowing river in Tibet. Siling Co is located at the end of the basin, and its water supplies are mainly in the form of precipitation, surface runoff, and glacier meltwater.

## 3. Data and Methods

### 3.1. Moderate Resolution Imaging Spectroradiometer (MODIS) Data

The MODIS instrument launched in December 1999 onboard the Earth Observing System Terra satellite was designed to observe and monitor Earth changes. MODIS has a swath of 2330 km, near daily global repeat coverage, onboard spectral calibration, and seven spectral bands with spatial resolutions of 250 m (bands 1 and 2) and 500 m (bands 3 to 7) [22]. The eight-day composite land surface reflectance (MOD09A1) data of MODIS from 2003 and 2013 (Table 1) were utilized in this study. The datasets include seven spectral bands at a spatial resolution of 500 m; these have been corrected for the effects of atmospheric gases, aerosols, and thin cirrus clouds [23]. The reflectance values of the near-infrared band (841 nm to 876 nm) of MOD09A1 were utilized to calculate the lake area of Siling Co with a threshold.

**Table 1:** Details of MODIS images.

Year	2003–2006	2007–2008	2009–2012	2013
Days	241	273	241	233
Month	August	September	August	August

### 3.2. Meteorological Data

Tibet only has a few meteorological stations. In this study, meteorological data from 1966 to 2013 obtained from the Xainza and Baingoin meteorological stations located near Siling Co were utilized. Such data include air temperature, precipitation, and pan evaporation data. Distinct cold and hot seasons of monthly mean air temperature in Siling Co lake area were observed. The mean air temperature from May to September was higher than 0 °C, whereas the air temperature in the other months was lower than 0 °C. Therefore, the months with air temperature higher than 0 °C were classified as the hot season, whereas the months with air temperature lower than 0 °C were classified as the cold season. The acquired lake MODIS data fell in August or September (Table 1). As such, the periods from October to December of last year and from January to April of the year were classified as belonging to the cold season, whereas the period from May to September was classified as belonging to the hot season.

Inclination rate was adopted to determine the perennial climate change trend, and the linear fitting method was used to derive the time series of climate factors. The linear regression equation can be expressed as:

***y*** = *a* + *bt* (1)

where *b* is the regression coefficient and *b* × 10 is the inclination rate representing the change trend of climate per 10 years.

### 3.3. Analysis Method of Delayed Response

Grey relational analysis (GRA) is adopted to investigate the delayed response of the Siling Co lake area to climate change. The fundamental principle of GRA focuses on analyzing and determining the effects of the comparison series on the reference series by investigating the geometric proximity among factors and calculating the grey relational grade (GRG) [15,21,24].

The reference series can be expressed as:

(2) $$X_{0}=\left[X_{0}\left(1\right),\;X_{0}\left(2\right),\;\ldots ,\;X_{0}\left(n\right)\right].$$

The comparison series can be expressed as:

(3) $$X_{i}=\left[X_{i}\left(1\right),\;X_{i}\left(2\right),\;\ldots ,\;X_{i}\left(n\right)\right],\;i=1,\;2,\;\ldots ,\;m.$$

The GRG between comparison series *X_i_* and reference series *X*_0_ can be expressed as:

(4) $$\gamma \left(X_{0},X_{i}\right)=\frac{1}{n}\sum_{k=1}^{n}r\left(x_{0}\left(k\right),x_{i}\left(k\right)\right),\;\;\text{γ}\left(X_{0},X_{i}\right)\in \left[0,1\right],$$

where:

$$\Delta_{0i}(k)=\left|x_{0}\left(k\right)-x_{i}\left(k\right)\right|,$$

$$x(\min)=\underset{i}{\min}\;\underset{k}{\min}\Delta_{0i}(k),$$

$$x(\max)=\underset{i}{\max}\;\underset{k}{\max}\Delta_{0i}(k),$$

(5) $$r\left(x_{0}\left(k\right),x_{i}\left(k\right)\right)=\frac{x(\min)+\text{ξ}x(\max)}{\Delta_{0i}(k)+\text{ξ}x(\max)},$$

where $r\left(x_{0}\left(k\right),x_{i}\left(k\right)\right)$ is the relational coefficient of *X_i_* to *X*_0_ at *k* and ξ is the grey resolution ratio with a value of 0.1. The GRG $\text{γ}\left(X_{0},X_{i}\right)$ reflects the mutual effects between series. When the value is close to 1, the influence of the comparison series on the reference series is more significant. The rank of GRG can be considered the significant rank of the reference series to the comparison series.

This research is based on the premise that lake area change generates a delayed response to regional climate change. With Siling Co lake area from 2003 to 2013 as the reference series (***X*_0_**), the comparison series adopted the normal factors (***X_i_***) in the Xainza and Baingoin meteorological stations from 1998 to 2013. Assuming that the delayed periods of lake area changes to climate changes is set as zero, one, two, three, four, or five years, GRA between climate (***X_i_***) and reference (***X*_0_**) factors was conducted according to the assumed delayed period (Table 2). The corresponding delayed period with the maximum GRG of the comparison series is the delayed response period of lake area changes to the corresponding climate change.

**Table 2:** Time list of reference and comparison series (Year).

Delayed Response Time	Reference Series (*X_0_*)	Comparison Series (*X_i_*)
0	2003–2013	2003–2013
1	2003–2013	2002–2012
2	2003–2013	2001–2011
3	2003–2013	2000–2010
4	2003–2013	1999–2009
5	2003–2013	1998–2008

## 4. Results and Discussions

### 4.1. Siling Co Lake Area Changes

**Figure 2:**
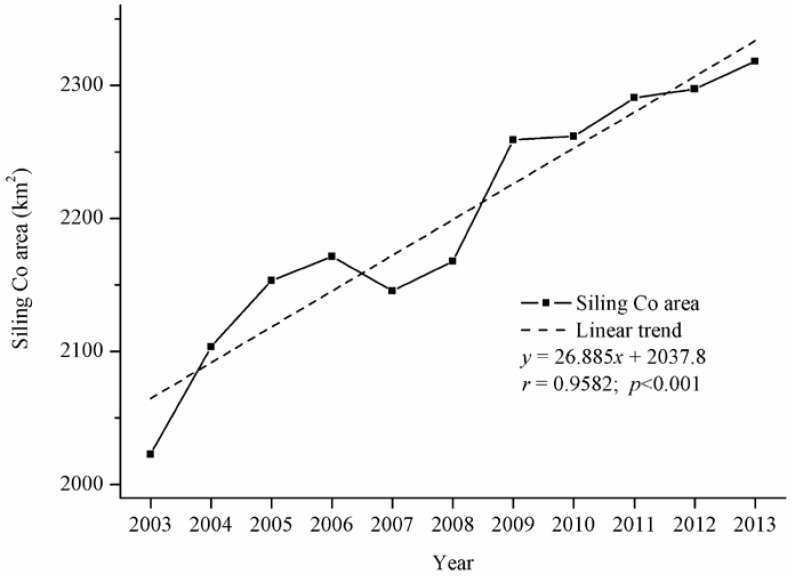
Annual changes in the lake area of Siling Co from 2003 to 2013.

The changes in Siling Co lake area from 2003 to 2013 can be classified into three periods (Figure 2 and Table 3), namely, a rapidly increasing period from 2003 to 2006, a decreasing and increasing period from 2006 to 2009, and a stably increasing period from 2009 to 2013. The lake area of Siling Co was 2022.73 km^2^ in 2003 and 2171.25 km^2^ in 2006. From 2003 to 2006, the increase in lake area was 148.52 km^2^, with a growth percentage of 7.34% and annual growth rate of 37.13 km^2^·year^−1^. From 2006 to 2009, the increase in lake area fluctuated. The lake area 25.75 km^2^ in 2007 was smaller than that in 2006, whereas the lake area in 2008 increased to the same area as in 2005. However, in 2009, the lake area increased significantly to 2259 km^2^. During this period, the growth rate of Siling Co lake area was 4.04%, with an annual growth rate of 21.93 km^2^·year^−1^. From 2009 to 2013, the lake area increased stably, with a small increase range (0.61%) and slow annual growth (11.8 km^2^·year^−1^). Overall, Siling Co lake area increased by 295.27 km^2^ with a growth rate of 26.84 km^2^·year^−1^ from 2003 to 2013.

**Table 3:** The lake area change of Siling Co from 2003 to 2013.

Year	Change (km^2^)	Change Rate (%)	Growth Rate (km^2^∙year^−1^)
2003–2006	148.52	7.34%	37.13
2006–2009	87.75	4.04%	21.94
2009–2013	59.00	2.61%	11.8
2003–2013	295.2713	14.60%	26.84

### 4.2. Changes in Climate Factors

The regional climate factor in the Siling Co area covering average air temperature, precipitation, and pan evaporation in hot and cold seasons were acquired from the Xainza and Baingoin meteorological stations.

#### 4.2.1. Air Temperature

**Figure 3:**
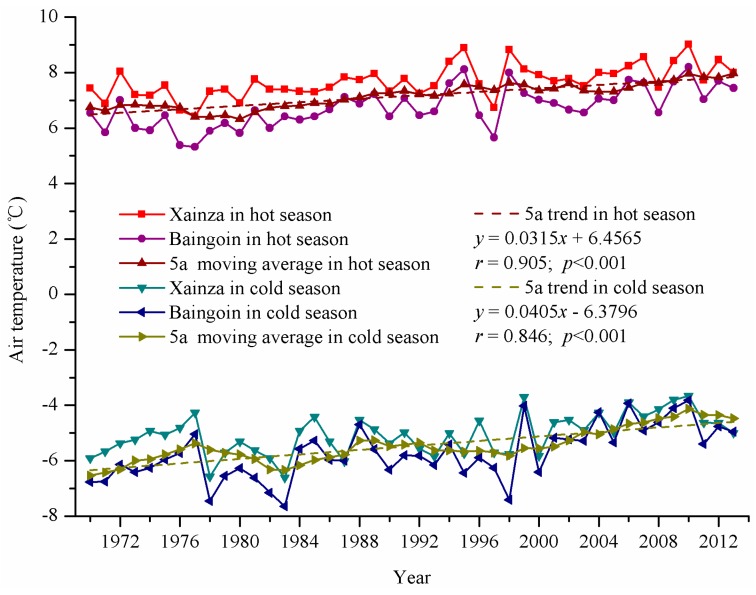
Annual mean air temperatures at Xainza and Baigoin stations from 1966 to 2013.

From 1966 to 2013, the increase in air temperature in Siling Co area fluctuated (Figure 3). The statistics show that the change trends in air temperature at the Xainza and Baingoin meteorological stations were consistent. However, the air temperature at the Xainza meteorological station was higher than that at the Baingoin meteorological station, particularly in the hot season. The inter-annual air temperature changes in the lake area manifested a stable increasing period from 1966 to 1994, with a higher growth rate of air temperature in the cold season (0.30 °C per ten years) than in the hot season (0.28 °C per ten years). The fluctuation period from 1995 to 1999 had an average air temperature amplitude of 2.31 °C in the hot season and 2.70 °C in the cold season (during this period, the air temperature in the hot and cold seasons was slightly lower than that in the previous period). In the rapidly increasing period from 2000 to 2013, the growth rate in the hot season was 0.56 °C per ten years, whereas the growth rate in the cold season was 0.14 °C per ten years.

Overall, for approximately 50 years (1966 to 2013), the increase trend in air temperature in the Siling Co area was evident, with increase trends of 0.32 °C per ten years in the hot season and 0.41 °C per ten years in the cold season. The increase trend in the cold season was greater than that in the hot season.

#### 4.2.2. Precipitation

**Figure 4:**
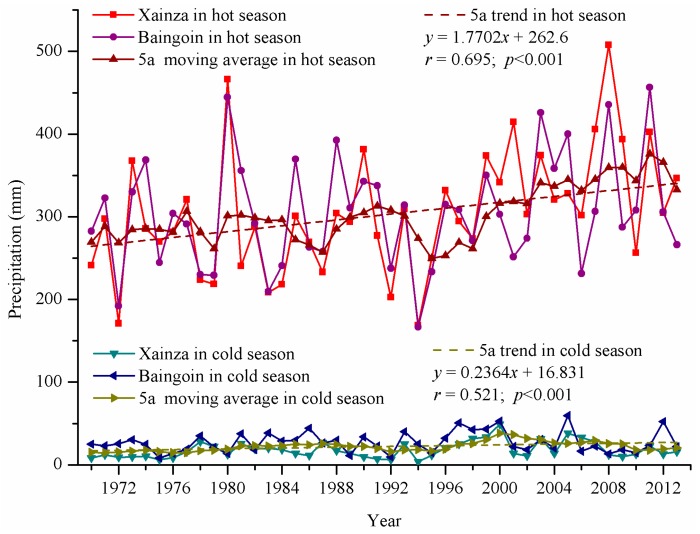
Annual precipitations at the Xainza and Baigoin stations from 1966 to 2013.

Precipitation in the Siling Co area had evident seasonal distribution characteristics, with precipitation in the hot season accounting for over 90% of the annual precipitation (Figure 4). The overall trends at the Xainza and Baingoin meteorological stations were almost the same. The precipitation in the cold season differed slightly. The precipitation in the hot season at the Xainza meteorological station from 1999 to 2002 and from 2006 to 2009 was greater than that at the Baingoin meteorological station. In the other periods, the precipitation in the hot season at the Baingoin meteorological station was greater than that at the Xainza meteorological station. From 1966 to 2013, regional precipitation presented a trend of “rapidly increasing-stably increasing-rapidly increasing”. From 1966 to 1980, the growth rate of precipitation in the hot season was 43.11 mm per ten years. From 1981 to 1993, precipitation in the hot season tended to increase stably, with a growth rate of 29.64 mm per ten years. From 1994 to 2013, precipitation in the hot season tended to rapidly increase, with a growth rate of 51.64 mm per ten years. In nearly 50 years, precipitation in the hot season in the Siling Co area tended to increase significantly, with a change rate of 17.70 mm per ten years. By contrast, precipitation in the cold season increased slightly, with a change rate of 2.36 mm per ten years only.

#### 4.2.3. Evaporation

**Figure 5:**
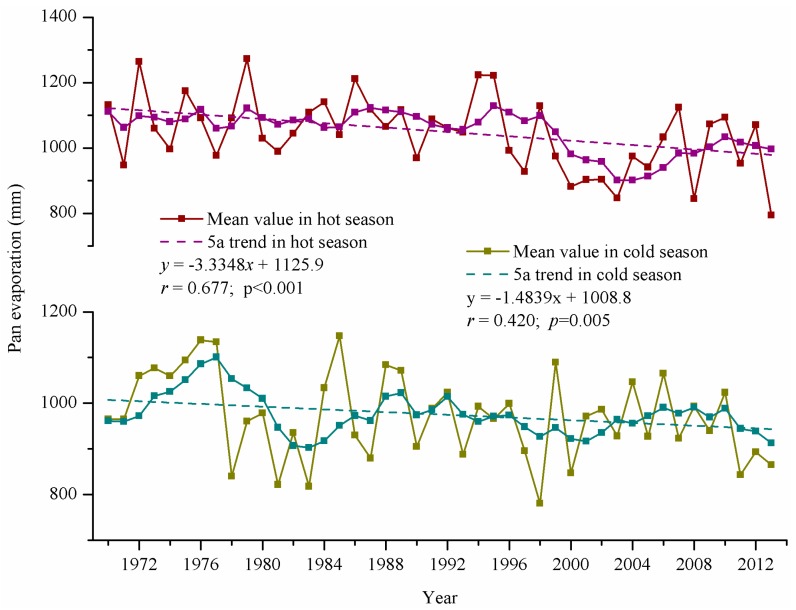
Annual pan evaporation in study area from 1966 to 2013.

Evaporation was analyzed from observation data on 20 cm small evaporation pans located at the Xainza and Baingoin meteorological stations. The water body in the evaporation pans was small such that the evaporation in the pans could not represent the actual evaporation of water bodies. However, the data would enable us to understand the change rules and trends of evaporation [25]. Evaporation in Siling Co area in hot and cold seasons was large. Evaporation in the hot season was larger than that in the cold season, with an annual average evaporation amount of approximately 2020.45 mm. The change in evaporation can be classified into two periods, namely, stably decreasing period from 1966 to 1993 and rapidly decreasing period from 1994 to 2013. From 1966 to 1993, the decrease rates of evaporation in the hot and cold seasons differed slightly, with 13.15 and 12.81 mm per ten years, respectively. However, from 1994 to 2013, evaporation tended to rapidly decrease, with decrease rates of 62.76 mm per ten years in the hot season and 19.07 mm per ten years in the cold season. Another characteristic during the period was the rapidly increasing evaporation in the cold season, with the same annual evaporation (944.86 mm) as that in the hot season (995.09 mm). In summary, evaporation in the study area from 1966 to 2013 decreased evidently, and the decrease rate in the hot season (33.35 mm per ten years) was greater than that in the cold season (14.84 mm per ten years) (Figure 5).

### 4.3. Analysis of Delayed Response

**Figure 6:**
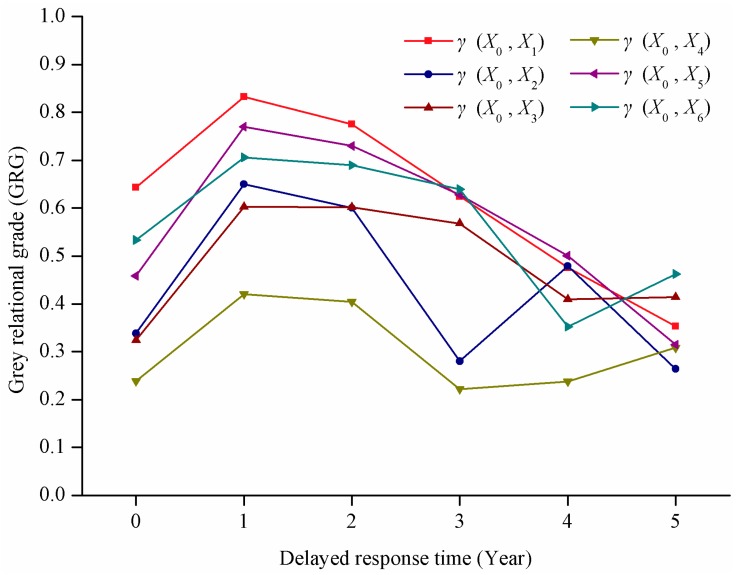
GRGs of delayed response time of the Siling Co lake area change to climate change.

Reference series ***X*_0_** and comparison series ***X_i_*** in the analysis of delayed response are shown in Table 4. Based on the delayed time sequence shown in Table 2, GRA was conducted (Table 5 and Figure 6). An evident delayed response of the Siling Co lake area to climate change was observed. The maximum GRG of various climate factors fell in the period of “delayed one year” followed by “delayed two years.” This finding indicates that delayed time is a period of time instead of an absolute value. In other words, the delayed response of the Siling Co lake area change to climate change is approximately one to two years.

**Table 4:** Series value of GRA.

*X*	Items	Factor Series
*X*_0_	Lake area of Siling Co	Reference series
*X*_1_	Hot season mean air temperature (°C)	Comparison series
*X*_2_	Cold season mean air temperature (°C)	
*X*_3_	Hot season precipitation (mm)	
*X*_4_	Cold season precipitation (mm)	
*X*_5_	Hot season evaporation (mm)	
*X*_6_	Cold season evaporation (mm)	

As a result of global climatic warming, the Qinghai-Tibetan Plateau glacier has tended to shrink in a comprehensive and accelerating way since the 1990s. According to the statistical comparison between the data of the second glacier inventory (remote sensing data were taken from 2006 to 2010) and the first inventory (remote sensing data were taken from 1950 to 1980) in China, the glacier area of the Qinghai-Tibetan Plateau within Chinese territory and its adjacent regions has retreated by 15% on average [26].There are 642 glaciers covering about 593.09 km^2^ in area and 36.37 km^3^ in ice volume in the Sling Co catchment [10]. From 1990 to 2011, the glacier area in the catchment has reduced by 34.76 km^2^, with a retreat ratio of 12.55% and an annual retreat rate of 1.66 km^2^·year^−1^ [14].

**Table 5:** GRGs between Siling Co lake area and climate factors in different delayed response time.

GRG	Delayed Response Time (Year)					
	0	1	2	3	4	5
γ(*X_0_*,*X_1_*)	0.6431	**0.8325**	**0.7755**	0.6244	0.4756	0.315
γ(*X_0_*,*X_2_*)	0.3384	**0.6501**	**0.6003**	0.2803	0.4791	0.2486
γ(*X_0_*,*X_3_*)	0.3239	**0.6028**	**0.602**	0.568	0.4098	0.4531
γ(*X_0_*,*X_4_*)	0.2389	**0.4206**	**0.4041**	0.222	0.238	0.2997
γ(*X_0_*,*X_5_*)	0.4586	**0.7698**	**0.7298**	0.628	0.5007	0.3148
γ(*X_0_*,*X_6_*)	0.5335	**0.7057**	**0.6896**	0.6392	0.3526	0.4627

**Notes:** γ(*X_0_*,*X_1_*), the GRG between hot season mean air temperature (°C) and Siling Co areas (km^2^); γ(*X_0_*,*X_2_*), the GRG between cold season mean air temperature (°C) and Siling Co areas (km^2^); γ(*X_0_*,*X_3_*), the GRG between hot season precipitation (mm) and Siling Co areas (km^2^); γ(*X_0_*,*X_4_*), the GRG between cold season precipitation (mm) and Siling Co areas (km^2^); γ(*X_0_*,*X_5_*), the GRG between hot season evaporation (mm) and Siling Co areas (km^2^); γ(*X_0_*,*X_6_*), the GRG between cold season evaporation (mm) and Siling Co areas (km^2^).

Permafrost in the Qinghai-Tibetan Plateau is also highly sensitive to climate change and has experienced significant temperature increases and widespread degradation during the last several decades. The active layer thickness of permafrost has increased 19 cm from 1990s to 2010s [27]. The ground ice melting is the main character of permafrost degradation [28]. During the process of permafrost degradation, some of the ground ice gradually melted into water to replenish underground water and increase its runoff. The complexity of hydrological processes makes it difficult to accurately evaluate the contribution of ground ice melting to the expansion of Siling Co. While, according to conservative estimation, the contribution of ground ice melting to regional hydrological cycling is over 1% [29].

The delayed response of Siling Co lake area changes to climate change has not been previously reported. There is no hydrological station in the Siling Co Catchment, so there is a lack of observed data on the glacier runoff and river runoff in this catchment. Here we use the research results of three inflow lakes in the Qinghai-Tibetan Plateau to explain the delayed response of Siling Co to climate. The lakes are Nam Co, Bankog Co and Qinghai Lake. The supply sources of Nam Co and Qinghai Lake are glacier runoff, river runoff and precipitation. Bankog Co has no supply of glacier runoff. Nam Co and Bankog Co have the similar climate background as Siling Co, and the linear distances to Siling Co are about 129 km and 7 km, respectively. Qinghai Lake is located in the northeast part of the Qinghai-Tibetan Plateau.

In recent years, Nam Co has also shown a trend of lake expansion and rising lake levels (Table 6). Zhu and Wu estimated that glacial melting from glacial surface reduction since 1970 contributed to 60% of the lake level increase [30]. The research on glacial mass balance carried out by Yao et al. also indicates that the glacial negative mass balance contributed to 50% of the Nam Co lake level rise [31].

**Table 6:** Characteristics of Siling Co and Nam Co.

Contents	Siling Co	Nam Co
Catchment area (km^2^)	50,106.2	10,680.4 [32]
Glacier area in catchment in 2007 (km^2^)	267	166.2 [30]
Lake area in 2013 (km^2^)	2320.12	2142.27
Lake area growth rate from 2001 to 2013 (km^2^·year^−1^)	29.95	12.84

**Figure 7:**
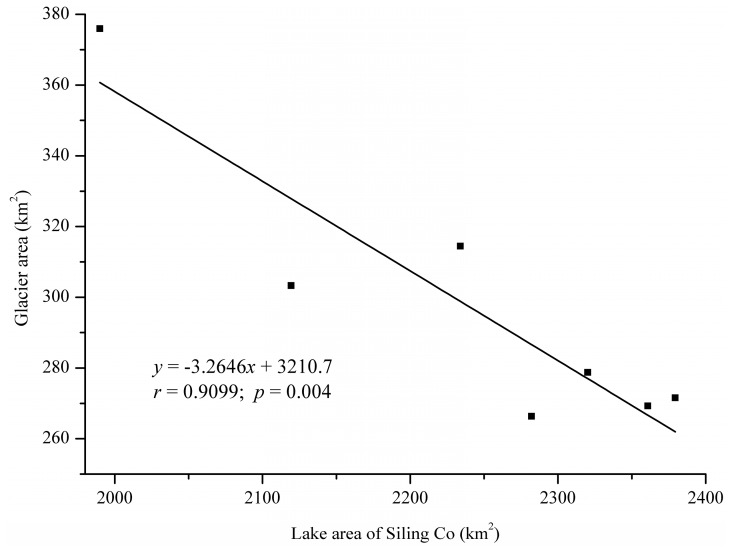
Scatter pot of the Siling Co lake area and glacier area.

The Sling Co lake level rose by 8.2 m while Bankog Co, which is not supplied by glacier runoff, only rose by 0.2 m from 2000 to 2010. Based on this, Meng *et al.* estimated that under the same climate background, glacier melting made Sling Co lake level rise by approximately 8 m during the 10 years from 2000 to 2010 [13]. In the meantime, the lake area of Siling Co has a significant linear correlation with the glacier area in the Siling Co catchment from 2001 to 2013 (Figure 7). Taking the contribution made by glacier runoff to the expansion of Nam Co as reference, it is conservatively estimated that glacier runoff contributes more than 60% to the area expansion of Siling Co.

The correlation analysis of meteorological data and lake level change in Qinghai Lake from 1961 to 2002 conducted by Li indicates that, in the past nearly 42 years, the lake level change has a one year delayed response to the inflow change, and the impact of evaporation in the previous year on lake level is greater than that of the year [33]. Moreover, the correlation analysis conducted by Bai of lake level change of Qinghai Lake and the precipitation in the previous year shows that there is a correlation coefficient of 0.64 [34]. It can be concluded that the lake level change of Qinghai Lake has a one year response to the precipitation change; thus the increase of precipitation this year would lead to the rise of lake level the next year.

This research finds that the response of the Siling Co lake area to meteorological factors lags by 1 to 2 years, which is similar to prior research findings [29,30]. Through GRA, the authors not only obtain the lag time of lake expansion response to meteorological factors, but also find the main sensitive factors. The GRG sequence of the factors within lag time is the sequence of sensitive factors. On the whole, the response of lake expansion to hot season air temperature comes first. Hot season evaporation, cold season evaporation, cold season air temperature, hot season precipitation and cold season precipitation follow it, respectively. The low response of Siling Co expansion to precipitation also shows that the accelerating glacier melting as a result of rising air temperature is the main factor leading to lake expansion in recent 10 years.

With global warming, glacier melting and permafrost degradation in Qinghai-Tibetan Plateau causes the increment of runoff and the rise of lake level, which brings about new problems to the regulation control and use of water resources. Therefore, we should establish more hydrometric stations to conduct research on hydrological processes and forecast the supply of glacier runoff to surface water and rivers, scientifically plan the use and development of water resource, and have a correct understanding of its regulation and control to surface water and regional climate.

## 5. Conclusions

Against the background of global warming, the climate in the Tibetan Plateau has become warmer and wetter. The lakes in the Tibetan Plateau exhibit significant changes. In the past 10 years, the lake area of Siling Co, which is located in the hinterland of the Tibetan Plateau, increased significantly. Based on MOD09A1 data from 2003 to 2013, the recent change rules in the Siling Co lake area were analyzed. By combining air temperature, precipitation, and evaporation data obtained from 1966 to 2013, we analyzed the delayed response of Siling Co lake area changes to climate changes through GRA. The main conclusions are as follows:

(1) The Siling Co lake area exhibited a rapid expansion trend from 2003 to 2013. The lake area had expanded by 295.27 km^2^ with a growth ratio of 14.6% and annual growth rate of 26.84 km^2^·year^−1^. From 2003 to 2006, the lake area expanded rapidly, with an annual growth rate of 37.13 km^2^·year^−1^.
(2) An increasing air temperature trend was evident in the Siling Co lake area from 1966 to 2013. The growth rate of air temperature in the hot season was 0.32 °C per ten years. By contrast, the growth rate of air temperature in the cold season was 0.41 °C per ten years, which is slightly greater than that in the hot season. Precipitation in the hot season increased evidently, with a growth rate of 17.70 mm per ten years. By contrast, precipitation in the cold season increased slightly. Pan evaporation exhibited evidently decreasing trends in hot and cold seasons, with rates of −33.35 mm and −14.84 mm per ten years, respectively. In the past 10 years, regional climate evidently became warmer and wetter.
(3) An evident delayed response of the Siling Co lake area change to climate change is observed, with a delay time of approximately one to two years. Overall, the response of lake area changes to air temperature in the hot season is the most significant, followed by hot season evaporation, cold season evaporation, cold season air temperature, and hot season precipitation. The response of lake area changes to precipitation in the cold season is the least significant.
